# Guest concentration, bias current, and temperature-dependent sign inversion of magneto-electroluminescence in thermally activated delayed fluorescence devices

**DOI:** 10.1038/srep44396

**Published:** 2017-03-15

**Authors:** Junquan Deng, Weiyao Jia, Yingbing Chen, Dongyu Liu, Yeqian Hu, Zuhong Xiong

**Affiliations:** 1 School of Physical Science and Technology, MOE Key Laboratory on Luminescence and Real-Time Analysis, Southwest University, Chongqing, 400715, P. R. China

## Abstract

Non-emissive triplet excited states in devices that undergo thermally activated delayed fluorescence (TADF) can be up-converted to singlet excited states via reverse intersystem crossing (RISC), which leads to an enhanced electroluminescence efficiency. Exciton-based fluorescence devices always exhibit a positive magneto-electroluminescence (MEL) because intersystem crossing (ISC) can be suppressed effectively by an external magnetic field. Conversely, TADF devices should exhibit a negative MEL because RISC is suppressed by the external magnetic field. Intriguingly, we observed a positive MEL in TADF devices. Moreover, the sign of the MEL was either positive or negative, and depended on experimental conditions, including doping concentration, current density and temperature. The MEL observed from our TADF devices demonstrated that ISC in the host material and RISC in the guest material coexisted. These competing processes were affected by the experimental conditions, which led to the sign change of the MEL. This work gives important insight into the energy transfer processes and the evolution of excited states in TADF devices.

Recently, significant research efforts have been focused on organic light-emitting diodes (OLEDs) that undergo thermally activated delayed fluorescence (TADF)^1^,^2^,^3^,^4^,^5^,^6^,^7^,^8^,^9^,^10^. TADF-based OLEDs can generate fluorescence with an internal quantum efficiency (IQE) of 100% without the need for rare metal complexes, which are commonly used in phosphorescent OLEDs^11^,^12^,^13^. The high IQE of TADF-based devices is the result of reverse intersystem crossing (RISC)^14^,^15^. RISC can cause the up-conversion of triplet excited states to produce singlet excited states, which can then undergo delayed fluorescence leading to a theoretical increase in the IQE from 25% to 100%^4^,^5^,^6^,^7^,^8^,^9^,^10^. For RISC to occur efficiently, a small energy gap between the singlet and triplet energy levels (Δ*E*_ST_) is required. Intramolecular charge transfer states (CTs) within molecules that contain spatially separated donor and acceptor moieties have a small exchange interaction because of the large interaction distance^16^,^17^,^18^. Thus, a small Δ*E*_ST_ can occur in molecules that contain CTs.

To avoid concentration quenching, the TADF material is usually incorporated into a device as a guest^15^,^16^,^17^,^18^,^19^. In energy-transfer dominated host-guest systems the host molecule generally serves as a recombination center where holes and electrons combine to form excited states before transferring their energy to the guest^20^. To the best of our knowledge, there are few reports that give a detailed understanding of the energy transfer process and evolution of excited states in TADF-based OLEDs. The magneto-electroluminescence (MEL) of an OLED is an effective tool to investigate the spin-dependent processes that occur within organic semiconducting materials and devices^21^,^22^,^23^,^24^. MEL is defined as: MEL = ΔEL/EL = [EL(*B*) − EL(0)]/EL(0), where EL(*B*) and EL(0) are the measured electroluminescence (EL) intensities with and without an applied external magnetic field (*B*), respectively. A small applied *B* can alter the population balance between singlet and triplet excited states in OLEDs and consequently change the total light intensity. Generally, the MEL in exciton-based OLEDs is positive with a full width at half maximum (FWHM) of several mT because the intersystem crossing (ISC) of polaron pairs is suppressed by the *B*^23^. Conversely, a negative MEL is observed in TADF-based OLEDs because any RISC from CTs is suppressed by the *B*, which has been described in the literature^25^,^26^,^27^.

In this paper, we report an unexpected positive MEL in a TADF-based device that contained CBP as host and 4CzTPN-Ph as guest. This device configuration was recently reported by Adachi *et al*.^15^ Moreover, the sign of the MEL became negative depending on experimental conditions, including doping concentration, current density and temperature. The MEL was positive when the concentration of 4CzTPN-Ph was lower than 5 wt% and became negative when the doping concentration was greater than 50 wt%. These observations were inconsistent with the argument that RISC is dominant in TADF devices. When the concentration of 4CzTPN-Ph was 50 wt%, the MEL tended to be negative under small injection currents (<100 μA), and positive at larger injection currents (~200 μA). Furthermore, the MEL tended to be negative at ambient temperature but positive at low temperature (20 K). These complicated behaviors were dictated by the energy transfer processes and evolution of the excited states between the host and guest materials.

## Results and Discussion

### Molecular structure and luminance properties

**Figure 1:**
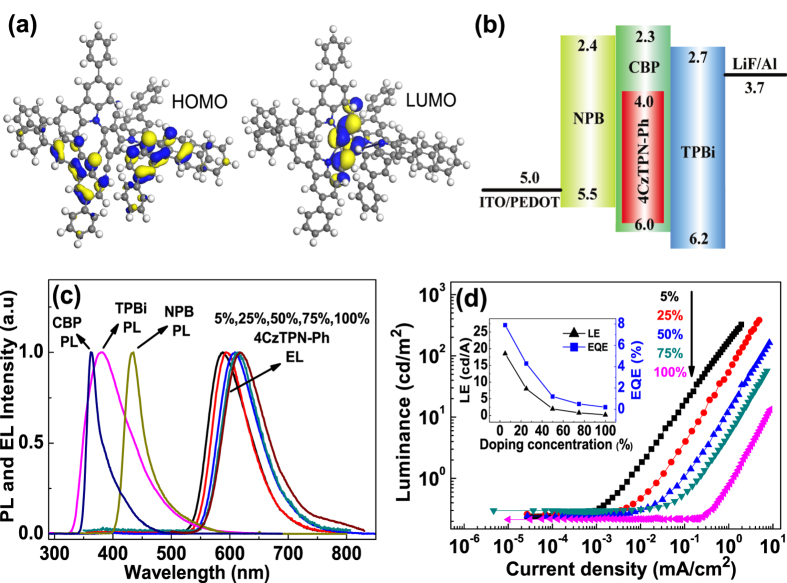
(**a**) HOMO and LUMO of 4CzTPN-Ph, calculated using DFT based on B3LYP. (**b**) Energy levels of TADF devices used in this study. (**c**) PL spectra of organic materials and EL spectra of TADF devices. (**d**) Luminance-current density curves of TADF devices. The inset shows the highest LE and EQE vs. doping concentration in TADF devices.

To ensure that a TADF material containing intra-molecular CT states has a sufficiently small Δ*E*_ST_, they are generally composed of donor and acceptor moieties^4^,^5^,^6^,^7^,^8^,^9^,^10^. The center of 4CzTPN-Ph contains a dicyanobenzene unit, which acts as an electron acceptor. The peripheral biphenyl carbazolyl units act as electron donors. The molecular geometry of 4CzTPN-Ph was optimized and the molecular orbitals were calculated using density functional theory (DFT) based on B3LYP^27^, as shown in Fig. 1(a). The highest occupied molecular orbital (HOMO) of 4CzTPN-Ph was localized on the carbazolyl moieties (donor), and its lowest unoccupied molecular orbital (LUMO) was centered on the dicyanobenzene moiety (acceptor). Consequently, the holes and electrons of the excited states are well separated in this molecule, which should lead to a small Δ*E*_ST_ and a large RISC rate constant^8^,^19^.

The structure and energy levels of the TADF-based device are shown in Fig. 1(b). PEDOT:PPS, NPB, CBP, 4CzTPN-Ph, and TPBi were used as hole injection layer, hole transport layer, host material, guest material, and electron transport layer, respectively. An exciton-based device (ITO/PEDOT:PSS/NPB/Alq_3_/LiF/Al) was fabricated for comparison. Normalized EL spectra of TADF devices with different doping concentrations, and normalized photoluminescence (PL) spectra of the organic materials at ambient temperature are shown in Fig. 1(c). CBP, TPBi and NPB all exhibited luminescence maxima between 350 and 420 nm. The EL emission maxima of the TADF-based device was located at 588 nm, which is close to the PL emission maxima of 4CzTPN-Ph (577 nm)^15^. Thus, the fluorescence of the host material (CBP) in the TADF-based device was completely quenched, which suggested that energy transfer between the guest and host molecules was efficient. As the concentration of the guest material (4CzTPN-Ph) was increased, the EL emission maxima exhibited a slight red shift, which was caused by the decrease in the polarity of 4CzTPN-Ph, as reported by Suh *et al*.^28^ The luminance of the TADF devices increased with increasing current density, as shown in Fig. 1(d). However, the luminance efficiency (LE) and external quantum efficiency (EQE) decreased with increasing doping concentration because of concentration quenching^29^,^30^ (inset, Fig. 1(d)). When the concentration of 4CzTPN-Ph was 5 wt%, the highest LE and EQE were 18.5 cd/A and 7.9%, respectively. This EQE was significantly higher than that of the exciton-based Alq_3_ device (EQE~2%). When the concentration of 4CzTPN-Ph was increased to 75 wt% the LE and EQE dropped to 0.8 cd/A and 0.5%, respectively. This indicated that concentration quenching within devices that are dense with TADF materials is significant.

### MEL response and mechanism of energy transfer within TADF devices

**Figure 2:**
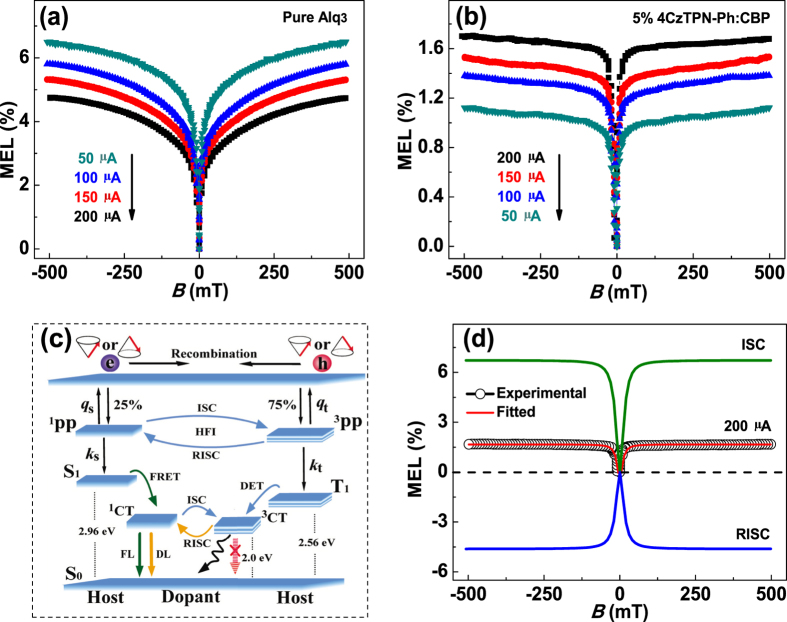
(**a**) MEL of the exciton-based device. (**b**) MEL of TADF device containing 5 wt% 4CzTPN-Ph. (**c**) Schematic diagram showing the processes that lead to electroluminescence in TADF devices. (**d**) MEL of TADF device containing 5 wt% 4CzTPN-Ph at 200 μA and its fitted line, which is the sum of the characteristic ISC and RISC MEL lines.

The MEL of exciton-based devices typically increases rapidly at low *B* strength and then slowly at high *B* strength, as shown in Fig. 2(a). When a device is subjected to a biased voltage the injected electrons and holes form polaron-pair (PP) states before excitons^23^. The Δ*E*_ST_ between singlet PP (^1^PP) and triplet PP (^3^PP) levels is comparable to the energy of the hyperfine interactions, which leads to ISC (^1^PP → ^3^PP) or RISC (^3^PP → ^1^PP)^31^,^32^,^33^. When an external *B* is applied, Zeeman splitting of the ^3^PP states suppress the ISC and leads to a positive MEL. As the *B* is increased further, the MEL saturates because of saturated suppression.

The spatial separation between the LUMO and HOMO levels in TADF molecules is much larger than that of the molecules used in exciton-based devices. This separation leads to significantly smaller Δ*E*_ST_ values between the singlet CT states (^1^CT) and triplet CT states (^3^CT), which in turn promotes RISC (^3^CT → ^1^CT). Therefore, the IQE is enhanced significantly, which leads to EL efficiencies that are several times higher in TADF devices than in exciton-based devices^4^,^5^,^6^,^7^,^8^,^9^,^10^. Therefore, if the external *B* suppresses RISC, this should result in a negative MEL^25^,^26^,^27^. However, a positive MEL was observed in our TADF device, as shown in Fig. 2(b).

Generally, the guest molecules in host-guest TADF devices are excited by energy transfer from the host molecules through Förster resonance energy transfer (FRET) and Dexter energy transfer (DET). FRET occurs at low doping concentrations between singlet excited states because it operates via an electrostatic dipole-dipole coupling. In contrast, DET normally occurs at higher doping concentrations between triplet excited states because it generally requires an electron exchange process^34^,^35^,^36^,^37^.

In our TADF device, the energy from the singlet exciton (S_1_) in CBP was transferred to the ^1^CT in 4CzTPN-Ph via FRET. The ^3^CT in 4CzTPN-Ph was excited by the triplet exciton (T_1_) in CBP through DET, and subsequently converted to the ^1^CT through RISC. The suppression of ISC between PPs (^1^PP → ^3^PP) in CBP by an external *B* should result in a positive MEL. However, the external *B* also suppressed RISC between CTs (^3^CT → ^1^CT), which led to a negative MEL. As ISC between PPs in the host material and RISC between CTs in the TADF material compete with each other, if the doping concentration of a TADF device is low, FRET will dominate. Therefore, if energy transfer from S_1_ to ^1^CT is dominant, a positive MEL will result. A schematic diagram showing the processes discussed above are presented in Fig. 2(c).

The experimental MEL curve (hollow black symbols) and its fitted line (red solid lines) at a current intensity of 200 μA within a *B* of ±500 mT are shown in Fig. 2(d) (the characteristic MEL of ISC and RISC are also shown). The MEL curves fitted perfectly to a Lorentzian function^25^,^26^, which can be expressed as:


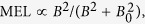


where the characteristic saturation field (*B*_0_) for ISC is ~10 mT. This value was consistent with the reported *B*_0_ for ISC^25^,^26^,^27^. At a current density of 5 mA/cm^2^, the MEL of the ISC process in exciton-based devices is generally ~6%, while the MEL in TADF devices is only ~1.5%. Therefore, RISC in our devices containing only 5 wt% TADF materials contributes ~−4.5% to the MEL.

The MEL curves from the TADF device (Fig. 2(b)) exhibited a similar line shape to those of the exciton-based device (Fig. 2(a)). However, the magnitude of the MEL from the TADF device increased with increasing injection current, while that of the exciton-based device showed the opposite trend. The EL of the exciton-based device increased faster than the ΔEL as the current was increased, which caused a decrease in the MEL (MEL = ΔEL/EL). Conversely, the ΔEL increased faster than the EL with increasing injection current in the TADF device. The mechanism for this opposing effect may be related to the influence of a high electric field on the lifetime of ^3^CT states^25^.

The lifetime of photo-generated excitons in most organic semiconductors has been estimated to be approximately 10^−7^~10^−9^ s^23^,^24^. Therefore, the lifetime of an exciton is greater than that of the spin flipping process (~10^−9^ s)^38^. The probability of exciton dissociation increases at higher electric fields, which leads to a dramatic decrease in the lifetime of an exciton. The dissociation of loosely bound CT states is more favorable because of their weak Coulomb attraction^39^,^40^. Therefore, ^3^CT states with shortened lifetimes cannot undergo RISC (^3^CT → ^1^CT) efficiently when a high voltage bias is applied. This then leads to MEL with larger magnitudes.

### MEL response of devices with different 4CzTPN-Ph concentrations

**Figure 3:**
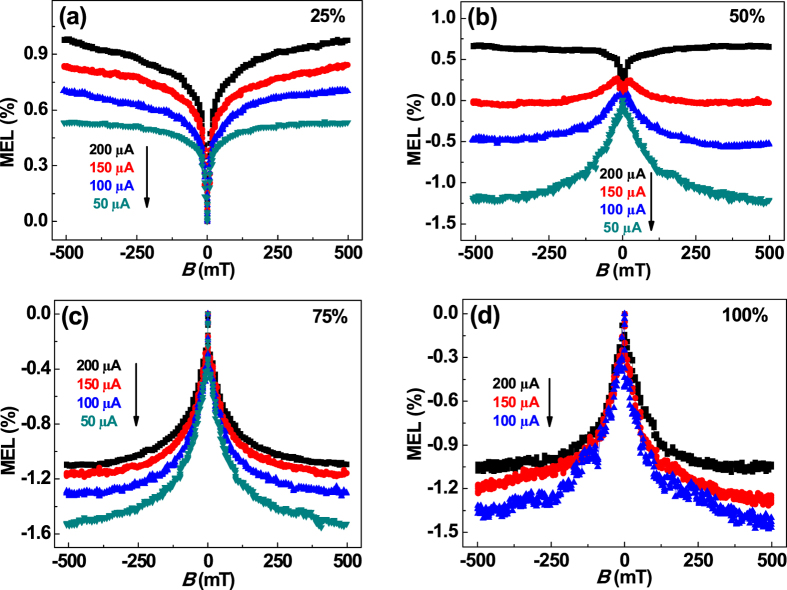
MEL of TADF devices containing (**a**) 25 wt%, (**b**) 50 wt%, (**c**) 75 wt%, and (**d**) 100 wt% 4CzTPN-Ph at different currents.

The concentration of the 4CzTPN-Ph in the TADF devices was altered to investigate our hypothesis. The concentration of guest molecule should affect the competition between the FRET and DET processes. A device that contained 25 wt% 4CzTPN-Ph exhibited a similar MEL line shape to that of a device containing 5 wt% (Fig. 3(a)), except that the magnitude of the MEL in the former device was approximately 0.7% smaller than that of the latter. This difference indicated that both DET and RISC (^3^CT → ^1^CT) were enhanced with increasing guest concentration. When the concentration of 4CzTPN-Ph was raised to 50 wt%, the sign of the MEL was either positive or negative, depending on the current intensity (Fig. 3(b)). In this case, the FRET and DET processes were comparable and competed with each other. RISC (^3^CT → ^1^CT) is dominant under low current conditions, and leads to a negative MEL line shape. As the current was increased, the strength of the electric field was enhanced and the ^3^CT lifetime was shortened, which diminished the RISC (^3^CT → ^1^CT) process.

When the concentration of 4CzTPN-Ph was greater than 75 wt%, DET was prominent, which caused the population of ^3^CT states to be larger than ^1^CT states. Under these conditions, RISC (^3^CT → ^1^CT) was dominant and caused the negative MEL line shape (Fig. 3(c) and (d)). However, when large currents were used, the lifetime of the ^3^CT state was lowered. Thus, RISC (^3^CT → ^1^CT) was weakened and the magnitude of the MEL decreased as the current was increased.

### MEL response of devices at different temperatures

**Figure 4:**
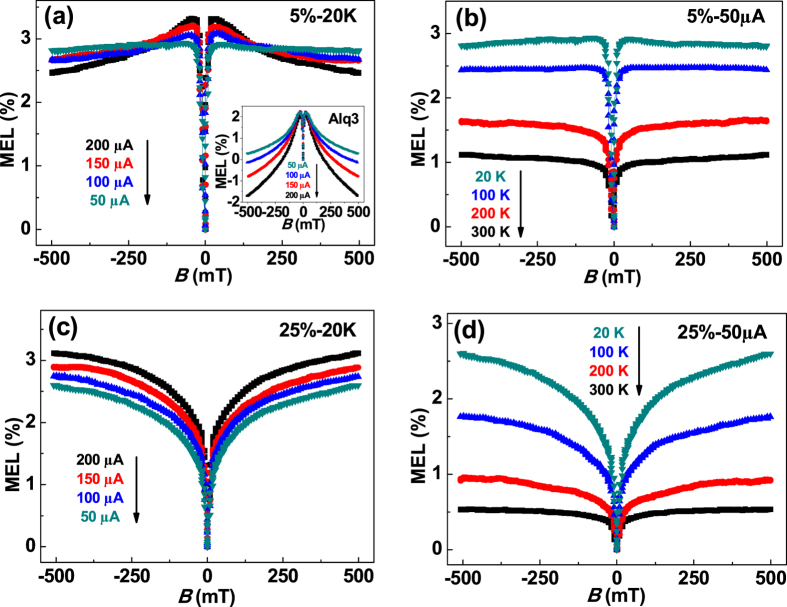
(**a**) MEL of a device containing 5 wt% 4CzTPN-Ph at different currents and a temperature of 20 K. The inset shows the MEL of the exciton-based device at different currents and a temperature of 20 K. (**b**) MEL of a device containing 5 wt% 4CzTPN-Ph at a current of 50 μA at different temperatures. (**c**) MEL of a device containing 25 wt% 4CzTPN-Ph at different currents and a temperature of 20 K. (**d**) MEL of a device containing 25 wt% 4CzTPN-Ph at a current of 50 μA at different temperatures.

RISC in TADF devices is an endothermic process that can be suppressed effectively at low temperatures^15^,^27^. Hence, if the negative MEL was caused by RISC (^3^CT → ^1^CT), it should become weaker at lower temperatures. Therefore, the MEL of the TADF device that contained 5 wt% 4CzTPN-Ph was measured at 20 K, as shown in Fig. 4(a). At low temperatures, the MEL was positive. Moreover, the exciton-based Alq_3_ device clearly exhibited triplet-triplet annihilation (TTA)^41^,^42^ (inset, Fig. 4(a)), while the TADF devices only exhibit weak TTA at low temperatures and high currents because TTA does not occur efficiently from CT states^43^. The effects of temperature on the MEL at a fixed current of 50 μA is shown in Fig. 4(b). The magnitude of the MEL increased from 1% to 3% with decreasing temperature, while that of the exciton-based device dropped from 6% to 2%. This indicated that RISC (^3^CT → ^1^CT) was suppressed at low temperature. When the doping concentration was increased to 25 wt% (Fig. 4(c)), the line shape of the MEL was characteristic of ISC without the presence of TTA, which was caused by the increased number of CT states. Moreover, the magnitude of the MEL was much larger at lower temperatures (Fig. 4(d)). This was consistent with a significant suppression of RISC from the CTs at lower temperature.

**Figure 5:**
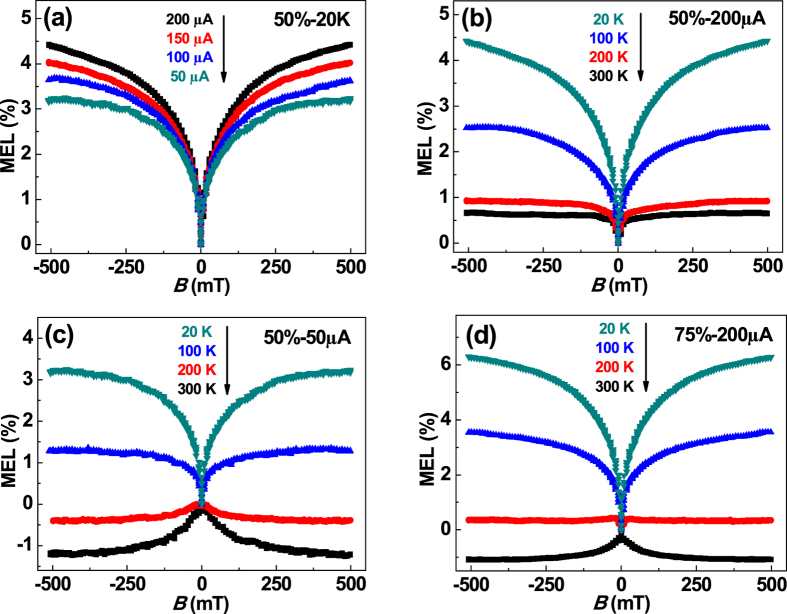
(**a**) MEL of a device containing 50 wt% 4CzTPN-Ph at different currents and a temperature of 20 K. (**b**) MEL of a device containing 50 wt% 4CzTPN-Ph at a current of 200 μA at different temperatures. (**c**) MEL of a device containing 50 wt% 4CzTPN-Ph at a current of 50 μA and different temperatures. (**d**) MEL of a device containing 75 wt% 4CzTPN-Ph at a current of 200 μA and different temperatures.

The MEL of a TADF device that contained 50 wt% 4CzTPN-Ph when measured at different currents at 20 K is shown in Fig. 5(a). The MEL of this device was also positive. At a current of 200 μA, the magnitude of the MEL increased from 0.6% to 4.5% as the temperature was decreased from 300 K to 20 K (Fig. 5(b)). This increase in magnitude was caused by a decrease in RISC from the CTs at low temperature, which is equivalent to an enhancement of the ISC process. Intriguingly, the sign of the MEL was affected by temperature. At a current of 50 μA, the MEL was negative at 300 K and positive at 20 K (Fig. 5(c)). This behavior was also observed in the devices that contained 75 wt% (Fig. 5(d)) and 100 wt% 4CzTPN-Ph, indicating that the negative MEL at ambient temperature was caused by RISC (^3^CT → ^1^CT).

### MEL response with different luminance efficiencies relating to temperature and concentration

**Figure 6:**
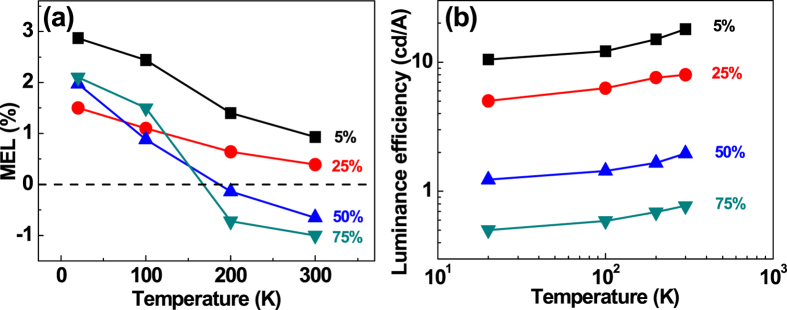
(**a**) The saturated MEL of devices containing 5 wt%, 25 wt%, 50 wt%, and 75 wt% 4CzTPN-Ph at different temperatures and a current of 50 μA. (**b**) The highest luminance efficiency of devices containing 5 wt%, 25 wt%, 50 wt%, and 75 wt% 4CzTPN-Ph at different temperatures.

The saturated MEL of the TADF devices within the hyperfine interaction range (<40 mT) are shown in Fig. 6(a). The hyperfine saturation value of the MEL increased with decreasing temperature, which was caused by a decrease in RISC from ^3^CT to ^1^CT at lower temperatures. Additionally, the magnitude of the MEL at 20 K was observed to increase as the doping concentration was increased from 25 wt% to 75 wt%, which indicated that the strength of RISC was proportional to the doping concentration. RISC is an endothermic process that can be suppressed at low temperatures. Thus, the highest EL efficiencies for the TADF devices decreased with decreasing temperature (Fig. 6(b)). Although RISC should improve the efficiency of a TADF device, concentration quenching can cause an overall lowering of device efficiency when TADF materials are present at high concentrations. This effect was observed in our devices, which indicated that concentration quenching was indeed dominant.

Time-resolved spectroscopy can be used to investigate the delayed fluorescence of TADF devices^15^,^24^. However, both RISC and TTA can generate delayed fluorescence and are difficult to distinguish using time-resolved spectroscopy. Fortunately, the MEL characteristics of RISC and TTA are clearly different and can be used to determine which process produced the delayed fluorescence. The MEL of RISC decreases monotonically (<40 mT) and quickly saturates as the *B* increases, while that of TTA increases slightly (<5 mT) and then drops with increasing *B* strength^25^. Additionally, ISC causes a MEL curve that is opposite to that of RISC. The MEL of our TADF devices showed characteristics of ISC and RISC but not TTA at ambient temperature. Thus, MEL allowed us to conclude that the delayed fluorescence from our TADF devices originated from RISC.

## Conclusion

In summary, the energy transfer processes and evolution of the excited states in TADF devices were investigated using MEL. This was done using OLEDs that had an energy transfer-dominated host-guest active layer. The host material acted as a recombination center for holes and electrons to generate excited states, followed by energy transfer to the guest. At low doping concentration, FRET was the dominant energy transfer mechanism. The TADF devices exhibited a positive MEL because ISC was dominant in the host material. However, the energy transfer of triplet excited states from the host to the guest was limited, which led to a low utilization of triplet excitons from the host material. Moreover, RISC was suppressed significantly at high current densities, which led to a decrease in the utilization of triplet CTs in the guest. At high doping concentrations, DET was dominant. The TADF devices exhibited a negative MEL because RISC was dominant in the guest. Although RISC should improve the efficiency of a device, the high concentrations of the TADF material can cause a decrease in efficiency because of concentration quenching. The results of this study indicated that the use of TADF materials as guests in devices may not result in improved efficiencies. However, if a TADF material was used as a host, both singlet and triplet excited states in the host material may be harvested efficiently via RISC and then subsequent FRET from the host to the guest. This hypothesis will be investigated in depth using MEL as a tool in future work.

## Methods

The TADF devices were fabricated with the following structure: ITO (120 nm)/poly(3,4-ethylenedioxythiophene):poly(styrenesulfonate) (PEDOT:PSS) (40 nm)/*N,N*′-*bis*(1-naphthalenyl)-*N,N*′-bisphenyl-(1,1′-biphenyl)-4,4′-diamine (NPB) (30 nm)/*x* wt% 2,3,5,6-*tetrakis*(3,6-diphenylcarbazol-9-yl)-1,4-dicyanobenzene (4CzTPN-Ph) (*x = *5, 25, 50, 75, 100): 4,4′-*bis*(carbazol-9-yl)biphenyl (CBP) (40 nm)/1,3,5-*tris*(1-phenyl-1H- benzimidazol-2-yl) benzene (TPBi) (50 nm)/LiF (1 nm)/Al (120 nm). The structure of the exciton-based OLED device that was used for comparison had the following structure: ITO (120 nm)/PEDOT:PSS (40 nm)/NPB (40 nm)/Alq_3_ (60 nm)/LiF (1 nm)/Al (120 nm). The PEDOT:PSS layer was spin-coated onto the ITO patterned glass substrate and then annealed for 10 min at 120 °C in a vacuum chamber. The layers consisting of small molecules were grown using an organic molecular beam deposition method under a base pressure of ~10^−6^ Pa. All devices were prepared with active area of 2 mm × 2 mm.

During measurement, the samples were mounted on the cold finger of a closed-cycle cryostat (Janis CCS-350S) that was located between two poles of an electromagnet (Lakeshore EM647). A Keithley 2400 SourceMeter was used to provide a constant voltage and measure the current. The brightness was determined using a magnetic insensitive silicon photodetector and recorded using a Keithley 2000 apparatus. The photoluminescence (PL) was measured with SpectraPro-2300i spectrometer with a laser excitation wavelength of 325 nm.

## Additional Information

**How to cite this article:** Deng, J. *et al*. Guest concentration, bias current, and temperature-dependent sign inversion of magneto-electroluminescence in thermally activated delayed fluorescence devices. *Sci. Rep.* **7**, 44396; doi: 10.1038/srep44396 (2017).

**Publisher's note:** Springer Nature remains neutral with regard to jurisdictional claims in published maps and institutional affiliations.
